# Premature mortality of epilepsy in low- and middle-income countries: A systematic review from the Mortality Task Force of the International League Against Epilepsy

**DOI:** 10.1111/epi.13603

**Published:** 2016-12-18

**Authors:** Francis Levira, David J. Thurman, Josemir W. Sander, W. Allen Hauser, Dale C. Hesdorffer, Honorati Masanja, Peter Odermatt, Giancarlo Logroscino, Charles R. Newton

**Affiliations:** * Ifakara Health Institute, Dar-es-Salaam, Tanzania; † Swiss Tropical and Public Health Institute, Basel, Switzerland; ‡ University of Basel, Basel, Switzerland; § Department of Neurology, Emory University, Atlanta, Georgia, U.S.A.; ¶ NIHR University College London Hospitals Biomedical Research Centre, UCL Institute of Neurology, London, United Kingdom; # Epilepsy Institute in The Netherlands (SEIN), Heemstede, The Netherlands; ** Sergievsky Center, Columbia University Medical Center, New York, New York, U.S.A.; †† University of di Bari Aldo Moro, Bari, Italy; ‡‡ Department of Neurosciences, Institute of Child Health, University College London, London, United Kingdom; §§ Department of Pediatrics, Muhimbili University of Health and Allied Sciences, Dar-es-Salaam, Tanzania; ¶¶ Department of Psychiatry, University of Oxford, Oxford, United Kingdom

**Keywords:** Seizures, Convulsions, Death, Case fatality, Developing countries, Resource-poor countries, Premature mortality

## Abstract

To determine the magnitude of risk factors and causes of premature mortality associated with epilepsy in low- and middle-income countries (LMICs). We conducted a systematic search of the literature reporting mortality and epilepsy in the World Bank-defined LMICs. We assessed the quality of the studies based on representativeness; ascertainment of cases, diagnosis, and mortality; and extracted data on standardized mortality ratios (SMRs) and mortality rates in people with epilepsy. We examined risk factors and causes of death. The annual mortality rate was estimated at 19.8 (range 9.7–45.1) deaths per 1,000 people with epilepsy with a weighted median SMR of 2.6 (range 1.3–7.2) among higher-quality population-based studies. Clinical cohort studies yielded 7.1 (range 1.6–25.1) deaths per 1,000 people. The weighted median SMRs were 5.0 in male and 4.5 in female patients; relatively higher SMRs within studies were measured in children and adolescents, those with symptomatic epilepsies, and those reporting less adherence to treatment. The main causes of death in people with epilepsy living in LMICs include those directly attributable to epilepsy, which yield a mean proportional mortality ratio (PMR) of 27.3% (range 5–75.5%) derived from population-based studies. These direct causes comprise status epilepticus, with reported PMRs ranging from 5 to 56.6%, and sudden unexpected death in epilepsy (SUDEP), with reported PMRs ranging from 1 to 18.9%. Important causes of mortality indirectly related to epilepsy include drowning, head injury, and burns. Epilepsy in LMICs has a significantly greater premature mortality, as in high-income countries, but in LMICs the excess mortality is more likely to be associated with causes attributable to lack of access to medical facilities such as status epilepticus, and preventable causes such as drowning, head injuries, and burns. This excess premature mortality could be substantially reduced with education about the risk of death and improved access to treatments, including AEDs.

Standardized mortality in people with epilepsy in high-income countries (HICs) is estimated to be up to 4–15 times higher than in the general population in community-based studies and selected high-risk populations, respectively.^1,2^ Comparable estimates of mortality in epilepsy in low- and middle-income countries (LMICs) are scarce, because vital registration of deaths is incomplete or absent in most countries, and many studies examining the risk of premature mortality in people with epilepsy in LMICs have methodologic limitations.

Worldwide, approximately 80% of persons with epilepsy live in LMICs.^3,4^ The estimated overall incidence of epilepsy is also higher in LMICs than in HICs (incidence rate ratio: 1.8, 95% confidence interval [CI] 1.3–2.5).^5^ The high prevalence and incidence of epilepsy in LMICs is most likely associated with the higher incidence of adverse perinatal events, head injuries, and parasitic infections.^4,6–10^

Some studies in LMICs^7,11,12^ have suggested that mortality in epilepsy is higher than in HICs.^2^ Higher mortality, whether comparing populations with and without epilepsy or populations of people with epilepsy in LMICs and HICs, can be described by relating age-specific mortality rates, most commonly expressed as standardized mortality ratios (SMRs; Table S1B). In effect, an elevated SMR in a population indicates excess *premature mortality*, that is, a higher proportion of deaths occurring at earlier ages, relative to the comparison population. Findings of relative excess mortality in LMICs may be attributed to the selection of studies in areas endemic with specific causes, selection of higher-risk cohorts to follow, or lack of access to comprehensive treatment.^6^ The epilepsy treatment gap (defined as the proportion of people with epilepsy who either have not accessed biomedical services or are not receiving treatment with antiepileptic drugs [AEDs] or are receiving inadequate treatment)^9^) is >75% in LMICs, compared with <10% in HICs.^10^ The large treatment gap may increase mortality from complications such as status epilepticus, accidents including burns and drowning, and sudden unexpected death in epilepsy (SUDEP).

We conducted a systematic review to estimate the magnitude of premature mortality associated with epilepsy in LMICs, and to identify the risk factors and causes of death among people with epilepsy. A companion review focuses on mortality in HICs.^13^

## Methods

### Literature search

We searched the Medline, EMBASE, and LILACS databases with terms in the following three categories:

1 epilepsy, seizure, or convulsions;
2 mortality, death, or SUDEP; and
3 low-income countries, middle-income countries, developing countries, resource-poor countries, Africa, Asia, China, India, “Latin America,” “Central America,” or “South America.”

We included only reports indexed with at least one term in each of the three categories, and restricted our search to reports on human subjects from LMICs as defined by the World Bank (low-income economies in 2014 were those with annual gross national incomes [GNIs] per capita of $1,045 or less, whereas middle-income economies were those with GNIs per capita ranging from $1,046 to $12,735. The country income categories for studies included in this report are indicated in Tables 1 and 2).^14^ The search period was from 1990 to February 28, 2014. We used the criteria for the diagnosis of epilepsy suggested by the International League Against Epilepsy (ILAE) for epidemiologic studies, originally in 1993^15^ and confirmed in 2010^16^ and 2011.^17^

**Figure 1:**
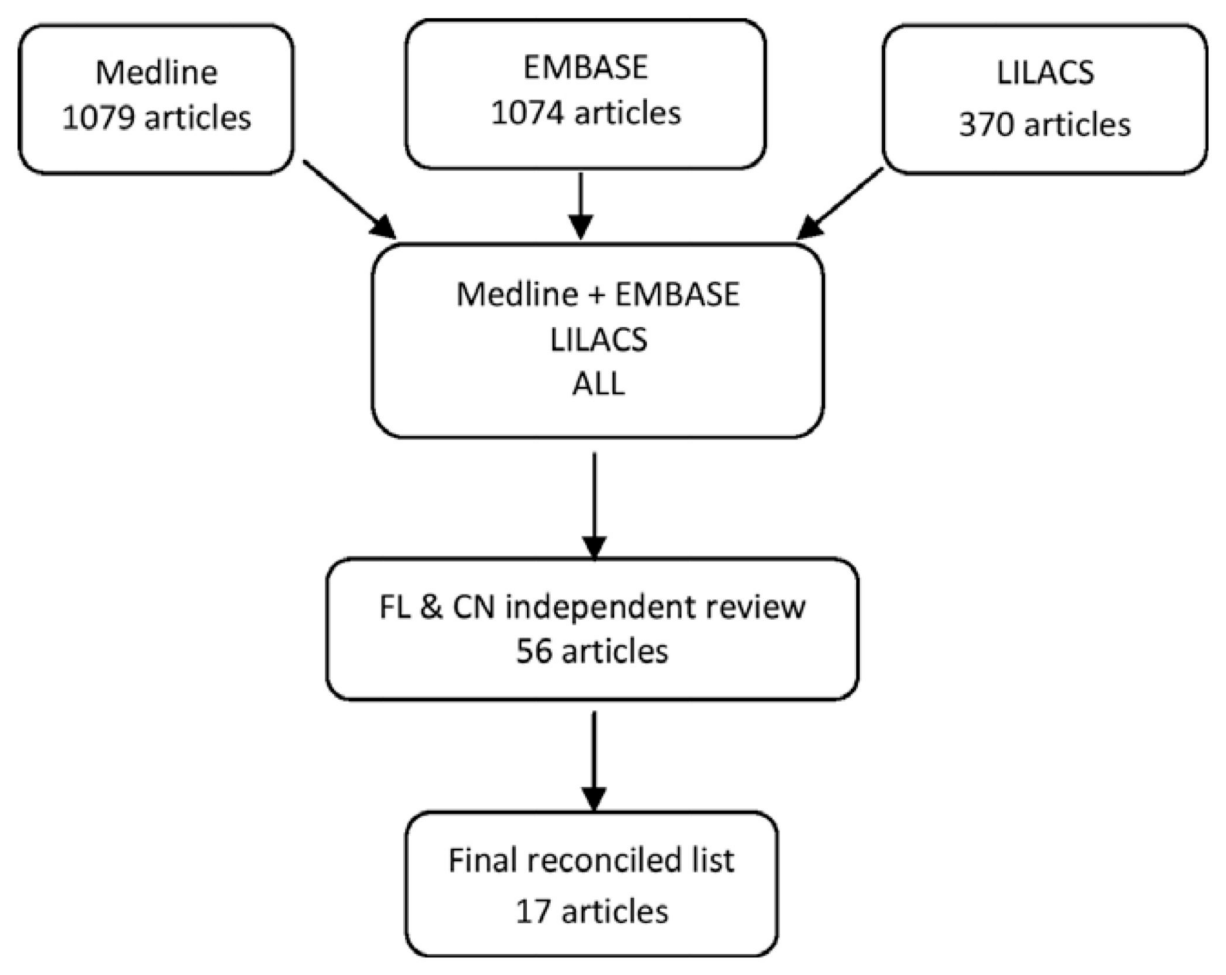
Summary results of search strategy. Flow diagram showing results of systematic literature search. Medline, EMBASE, and LILACS databases were used to search for scientific articles of studies of mortality associated with epilepsy between 1990 and 2014. The search used medical subject headings associated with epilepsy and its manifestation, mortality, and geographic location restricted to low- and middle-income countries. Authors FL and CN independently evaluated citations of the search output by reading title and abstract and arrived at a total of 56 articles. Through the inclusion criteria developed, a total of 17 articles were finally included in the systematic review. *Epilepsia* © ILAE

Two reviewers (FL and CRN) evaluated the retrieved citations in a two-stage process. In the first stage they independently reviewed the titles and available abstracts to identify potentially relevant reports meriting full review (Fig. 1). The reviewers compared their selections and resolved the list of publications to arrive at a single list for the second stage of analysis. In the second stage they reviewed the full papers and assessed whether the articles met the inclusion criteria below. For those meeting these criteria, they extracted the measures of mortality, risk factors, and causes of death.

### Inclusion criteria

Original reports of mortality among people with epilepsy in LMICs, derived from general populations, clinical cohorts (hospital- or treatment program-based), and case–control studies, were included. Studies that did not report quantitative estimates of mortality in epilepsy were excluded.

### Data extraction

Data on the epilepsy were extracted according to ILAE guidelines, in particular age at onset, seizure type, and the underlying etiology.^17^ Age at onset, that is, first occurrence of unprovoked seizure, helps identify epilepsy syndromes. Three main categories of *epilepsy/seizure type* as classified by ILAE (generalized, focal, and undetermined) were identified.^16,17^ The ILAE proposed three main categories of etiology: genetic, structural/metabolic, and unknown causes.^16,17^ Epilepsy etiology is classified as genetic when genetic defects are the known or presumed to be the prime cause of the disease (including epilepsies formerly known as idiopathic). Structural/metabolic causes (formerly known as symptomatic epilepsies) are considered when a structural lesion or metabolic condition is known to predispose to epilepsy. It was expected that some studies would still report earlier epilepsy etiology categories as idiopathic, symptomatic, and cryptogenic (now termed unknown).^18^

Estimates of mortality were extracted from the measures reported in the articles that included case fatality ratio (CFR), proportional mortality ratio (PMR), mortality rate (MR), and SMR according to standard definitions (Table S1B).^19^ Deaths were categorized as occurring: (1) as a direct consequence of epilepsy or seizures (i.e., status epilepticus, or SUDEP); (2) as indirect causes (i.e., accidents due seizures—such as falls, burns, or drowning—or drug reactions to AEDs); (3) as unrelated to epilepsy; or (4) as undetermined (i.e., cause of death not ascertained). The definitions and classification of SUDEP are mainly consistent with the earlier recommendations,^20^ which have been updated since publication of most the studies we reviewed.^21^

### Quality of studies

The reviewers employed quality assessment criteria for studies of mortality in epilepsy that included the following five elements classified by ILAE’s Commission on Epidemiology Task Force on the Burden of Mortality (Table S1A). These address the most important design features of epidemiologic studies of epilepsy, which we employed in preference to less-specific conventional evaluation checklists proposed for observational studies.^22^

1 Representativeness of the study population: Provides the basis for generalizability of the study findings. Studies conducted in clinical settings or untreated populations may not be representative of entire populations of people with epilepsy.
2 Accuracy of diagnosis of epilepsy: Evaluates methods employed to diagnose cases of epilepsy; if quantifiable, can be expressed as a positive predictive value. The use of the case definition provided by ILAE (at least two unprovoked seizures) and diagnosis by trained neurologists is a reference standard that may reduce the number of false positives and negatives.
3 Epilepsy case ascertainment: Evaluates completeness (sensitivity) of methods employed to screen the population for cases of epilepsy. This is important in population-based studies of epilepsy prevalence or incidence. A door-to-door survey is thought to generate an optimum number of cases in screening of a community.
4 Mortality ascertainment: Evaluates the completeness of identifying death occurrence in a cohort or population of people with epilepsy. This depends on the proportion of people with epilepsy followed until death or end of the study. Short follow-up of individuals, high migration patterns, and loss to follow-up are critical issues in establishing mortality rates.
5 Accuracy of cause of death: Evaluates the validity of cause-of-death determinations, especially for causes of interest associated with epilepsy. Accurate determination of the cause of death is essential to determine the proportion of deaths directly or indirectly related to epilepsy. Autopsies are the reference standard, but are rarely performed in LMICs. Verbal autopsies are most commonly used in LMICs.^23–25^

A scale with five items was developed to score the quality of studies with respect to each quality measure listed in the preceding. The scoring of each item was generated from 4 to 5 subitems, each with 5 points, with a total score ranging from 0 to 20 (Table S1A). The total score of the quality was the sum of scores of each quality item, with 100 representing the highest quality.

### Statistical analysis

Total, median, and range were used to generate summary measures of mortality across studies. We disaggregated the estimates by study designs (population-based vs. clinical cohort), sex, type of seizure, etiology, and other risk factors. Summary estimates were also identified as reported, whether SMRs, PMRs, or MRs. If not reported as such, we calculated CFRs and MRs when the reports provided sufficient information to allow this. Standard definition of summary estimates can be found in Table S1B.

We also examined heterogeneity statistics, which measure the extent to which SMRs vary between studies, including the I^2^ statistic, which is the percentage of between-study heterogeneity that is explained by variability in the exposure effect on mortality relative to sampling error.^26^ Heterogeneity statistics determine whether the SMR from individual studies can be combined.

## Results

### Search results

Results of the systematic search are provided in Figure 1. A total of 17 articles met inclusion criteria, among which one article included reports from four sites.^27^ and two articles reported separate findings from the same cohort.^28,29^ Thus, the articles contained 12 reports of findings from population-based cohorts^7,8,27,29–35^ and eight reports from clinical cohorts.^12,27,36–41^ South America provided eight studies,^8,27,34,36–40^ Asia six studies,^7,27–29,33,41^ and Africa six studies.^12,27,30–32,35^ Nine studies were conducted in rural populations only,^12,27,29–35^ six in urban only,^7,8,27,38,41^ and five in both urban and rural populations.^27,36,37,39,40^

### Quality of studies

Table S2 provides summary characteristics of population-based studies, all of which included all ages. Population-based studies were mainly of good quality, where 7 of 12 studies had a quality score of ≥80% (Table 2).^7,8,27,30,31,34,35^ Door-to-door surveys were implemented in most studies, where neurologists made diagnoses of epilepsy. Over 85% of deaths were estimated to be captured in these cohorts, and verbal autopsy was used to diagnose causes of death. Most studies used an operational definition of epilepsy as defined by ILAE in the diagnosis of epilepsy, although studies of three populations were restricted to active convulsive epilepsies.^29,33,35^

Clinical cohort studies were of low quality (≤50%), largely due to poor sample representativeness of the general population (Table S3). There was insufficient information published in most studies to determine the basis of the diagnosis of epilepsy. Individuals in clinical cohorts often had epilepsy described as intractable or refractory.^36,38,39^ Cohorts of children with severe forms of epilepsy were also enrolled in several studies.^37–40^ In healthcare settings, causes of death were available from medical records, death certificates, and verbal autopsy for deaths occurring in the communities.

### Mortality

From seven population-based studies with quality scores ≥80% (Table 1),^7,8,27,30,31,34,35^ the median annual mortality rate was 19.8 deaths per 1,000 people with epilepsy (range 9.7–45.1), with a weighted median SMR of 2.6 (range 1.3–7.2) and an overall CFR of 8.1% (range 3.3–31.6%). The weighted mean follow-up period was 5.8 years (range 1.5–10), with 6,665 person-years observation.

Eight clinical cohorts (Table 2)^12,27,36–41^ in sum enrolled substantially larger numbers of people with epilepsy compared to higher-quality population-based studies. There were 3,856 people with epilepsy enrolled across these cohorts, of which 88.3% were followed to the end of the studies. The weighted mean follow-up period was 12.4 years (range 3–30), during which 240 deaths were observed, with a pooled annual mortality rate of 7.1 (range 1.6–25.1) deaths per 1,000 people with epilepsy. Two of the eight studies reported SMRs of 3.2 and 6.3.^27,37^ The overall CFR in these clinical cohort studies was 5.4% (range 1.3–75.3%).

### Mortality risk by age

**Figure 2:**
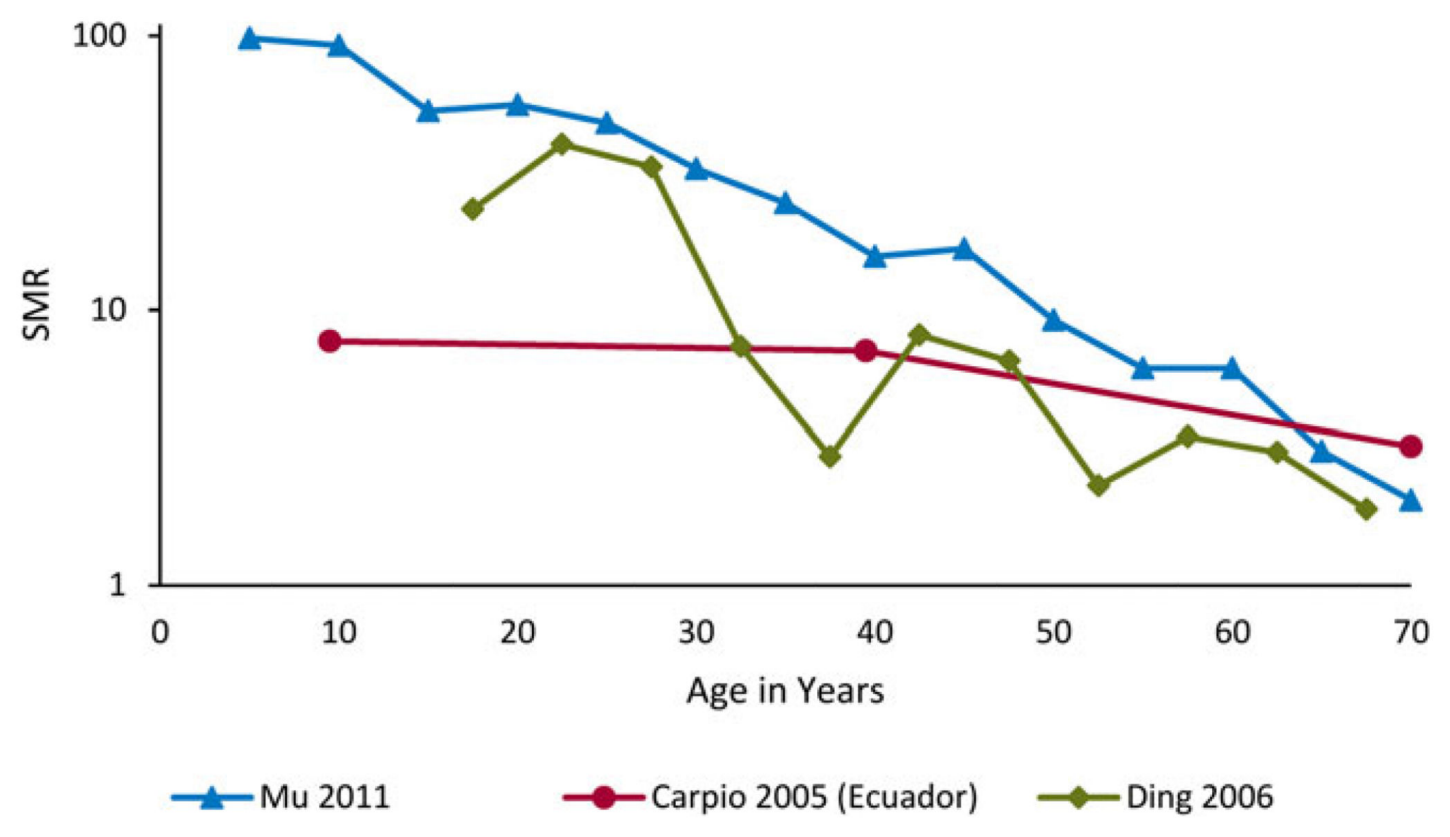
Mortality in epilepsy by age at death. Estimates of mortality rate by age. SMR stands for standardized mortality ratio; ratio of age standardized mortality rate in epilepsy and general population. SMR >1 represents excess mortality in epilepsy compared to the general population. *Epilepsia* © ILAE

Figure 2 summarizes three population-based studies reporting SMRs among people with epilepsy by age of death.^27,28,33^ Overall, these showed the highest SMRs in the youngest age groups, declining markedly after young adulthood, with a continuing decline with increasing age. We found insufficient data to characterize the risk of mortality by age of epilepsy onset.

### Mortality risk by sex

Figure S1 summarizes mortality estimates for males and females from six population-based studies and six clinical cohorts. Of five studies^27,28,31,33,35^ comparing SMRs, two reported higher values among males, with a weighted median SMR of 5.0 for males compared to 4.5 for females (Fig. S1A). Most studies reporting PMRs showed higher mortality in males than in females, with weighted medians of 62% and 38%, respectively (Fig. S1B).

### Cause-specific mortality in epilepsy

Table 3 presents eight population-based studies that reported cause-specific proportional mortality rates in those with epilepsy.^7,27,29,31–35^ Among people with epilepsy, the weighted median PMR for all causes of death directly or indirectly attributable to epilepsy was 47%. Direct causes comprised status epilepticus (median PMR 13%), and possible or probable SUDEP (median PMR 13%; Table S4). Indirect causes included accidents (falls, road traffic, drowning, and burns; Table S5). Among accidents, the median PMR was 15% (range 3.3–45%) for drowning,^7,28,31–33^ and 7.5% for road traffic accidents^7,28,33,35^; the remaining causes did not have sufficient data for a summary (Table S5). Among these population studies, the median sum of all listed accident-related PMRs was 36% of all deaths in people with epilepsy. Other important causes of death in these populations that were not attributable to epilepsy included cerebrovascular diseases, tuberculosis, malaria, heart disease, and cancer (Table S6).

Six clinical cohort studies reported causes of death as PMRs for individuals with epilepsy (Table 3).^12,27,36–39^ The median PMR for direct and indirect causes of death attributable to epilepsy was 39.3% and 24%, respectively. Median PMRs for deaths due to status epilepticus and SUDEP in clinical cohorts were 14.8% and 11.1%, respectively (Table S4). Data regarding causes of death indirectly attributable to epilepsy and causes not attributable to epilepsy were not sufficient to enable generalization.

### Mortality risk by seizure type or frequency

Mortality risk by seizure (or epilepsy) type was reported from two population-based^8,34^ and two clinical cohorts^36,41^ in which median PMRs were consistently higher for focal epilepsy (population-based—55% and clinical cohort—73%) than for generalized epilepsy (population-based—39% and clinical cohort—26%; Table S7). Two studies reported increased mortality among people with epilepsy, with a higher frequency of seizures^31,40^ (Table S8).

### Risk by duration of epilepsy

Two studies reported on mortality by duration of epilepsy^31,35^ (Table S9). A study in rural Kenya reported the highest mortality rate (45 per 1,000 person-years) for people with epilepsy duration of <1 year. In Uganda, SMRs were 8.6 (95% CI 4.5–16.5) for people with epilepsy of duration <5 years, 3.6 (1.1–11.4) for those of epilepsy duration 5–9 years, and 23.8 (8.9–65.5) for those of epilepsy duration 10–14 years.

### Risk by epilepsy etiology

Symptomatic epilepsies appeared to have higher mortality rates compared to cryptogenic epilepsy,^8,34,36,37^ although only two studies provided direct comparisons^34,36^ (Table S10).

### Risk by treatment adherence

Five population-based studies reported mortality risk by treatment adherence,^31–35^ treatment allocation,^32^ time since last treatment,^34^ and dose allocation^33^ (Table S11). In Kenya, mortality rates were three times higher for individuals who did not adhere to AED treatment than for those who adhered to treatment (mortality rate ratio 3.37, 95% CI 1.84–6.16).^35^ In Uganda, the SMR was 7.4 (95% CI: 3.9–14.1) for those with self-reported good AED adherence compared to 8.0 (95% CI: 3.8–16.4) for those with poor adherence.^31^ In Cameroon, treatment with AEDs alone was associated with 14% of deaths, compared to 27% in those using AEDs and traditional medicine.^32^ In one Chinese study, people receiving higher daily doses of phenobarbital (201–240 mg) had a lower mortality of 9% compared to those receiving medium doses (90–180 mg) with 44% mortality, and low doses (30–90 mg) with mortality of 47%.^33^ In a Bolivian study, recent access to AEDs was associated with lower mortality than absence of treatment for an extended period.^34^

### Meta-analysis

Meta-analysis revealed high heterogeneity across population-based studies as well as clinical cohort studies, and among high- and low-quality studies as well as short and long follow-up studies (Figs. S2 and S3). Accordingly, a formal meta-analytic synthesis was not attempted.

## Discussion

### Summary findings

This study provides evidence of higher premature mortality in people with epilepsy than in the general populations of LMICs, based on systematic reviews of epidemiologic studies accrued over 25 years. The number of quality studies found is low—especially considering this length of time and the large majority of the world population residing in LMICs—and points to the need for much further research. Nevertheless, these limited studies indicate that the main causes of death in people with epilepsy in LMICs are those directly attributable to epilepsy, that is, status epilepticus and SUDEP, and indirectly related to epilepsy, that is, drowning, head injury, and burns. The burden of premature mortality related to epilepsy appears higher in LMICs than in HICs. Unmet healthcare needs and lack of capacity in managing seizures may be the main reasons for the mortality gap between HICs and LMICs.

### Interpretation

Recognizing noteworthy earlier reviews of mortality associated with epilepsy specific to Africa^11^ and Latin America,^42^ our systematic review now examines a broader range of studies from LMICs across Africa, Asia, and South America. The incidence of epilepsy varies substantially across regions comprising LMICs and is higher in regions with high incidence of encephalitis and meningitis and with endemic parasitic infections such as malaria and neurocysticercosis. The availability of basic public health services as well as specialized health care for epilepsy also varies considerably among LMICs.^43^ Both factors may strongly influence epilepsy-related mortality. Thus, any generalizations drawn from the modest number of studies we have reviewed should be applied to other LMIC regions with great caution.

Despite their limitations, these data from LMICs provide clear evidence that the burden of premature mortality, as measured by SMRs, is higher among people with epilepsy than in corresponding general populations. Among the regions represented, the risk of premature mortality in people with epilepsy is particularly high in Africa, where the reported annual mortality rates in population-based studies range from 22.2 to 45.1 per 1,000 people with epilepsy,^27,30–32,35^ among which are reported SMRs of 6.5 and 7.2.^31,35^

SMRs are especially high in children and young adults with epilepsy. This finding might be explained in part by: (1) the comparatively lower mortality rates in general populations of young age, and (2) the increased mortality of recent-onset epilepsy associated with symptomatic or structural/metabolic etiologies, which occur with a substantial proportion of young cases.^1^ In HICs, higher SMRs have likewise been found in studies with age strata including children (range 6.4–8.5).^13^ Only one clinical cohort study in Ecuador^27^ reported an SMR (9.5) in a stratum similar to those of the HIC studies. Two other studies of lower quality from rural China reported extremely high SMRs among children, but with wide CIs.^28,33^

The burden of premature mortality among people with epilepsy in LMICs appears to be somewhat higher in males, as indicated by the PMRs from several studies. This could possibly reflect a higher incidence among males of symptomatic epilepsies (especially from traumatic brain injury), which have an increased mortality risk. In lower-income regions such as Africa, brain injuries commonly arise from vehicular collisions, occupational accidents, sports, and violence,^44^ which may be more frequent among males. The increased risk of premature mortality among males with epilepsy could also reflect mortality during hazardous occupations that carry an increased risk of drowning, falls, or other fatal injuries consequent to seizures.

These data also indicate that among the most important causes of death are those directly related to epilepsy, in particular status epilepticus, previously recognized to have a high CFR in LMICs.^45^ The proportion of deaths from status epilepticus appears substantially higher in LMICs compared to HICs.^13^ This difference might be explained in part by better access to AED treatment, better management of seizures, and better access to prompt treatment for prolonged seizures in HICs compared to LMICs.

SUDEP was identified as another cause related directly to epilepsy in several LMIC studies. The estimated proportions must, however, be viewed with caution, as causes of death were seldom diagnosed through postmortem evaluations, and diagnoses of SUDEP made by verbal autopsy or clinical history may be inaccurate.^18^ Nevertheless, many of the risk factors associated with SUDEP are more common in LMICs than in HICs, for example, structural causes of the epilepsy and frequent seizures, and thus SUDEP, may represent a greater burden in these regions than indicated by some studies. Specific studies to examine SUDEP occurrence in LMIC populations are needed.

In comparison to HICs, a higher proportion of people with epilepsy in many LMIC regions also die due to indirect causes, especially accidents. Many of these causes, for example, drowning and burns, are potentially preventable through education and safety measures. For other causes, for example, seizure-caused fatal injuries incurred during work or while driving a vehicle, further studies are needed to understand the frequency and circumstances of these injuries and to identify appropriate prevention measures that are specific to different localities.

### Comparing mortality in LMICs and HICs

Overall, the relative premature mortality risk associated with epilepsy in LMICs appears higher than the risk reported in HICs. In the best-quality LMIC studies, the weighted median SMR of 2.6 modestly exceeds the corresponding value of 2.2 in HIC.^13^ Such comparisons of SMRs between studies are, however, fraught with potential error, especially given differing age distributions among study populations (with generally larger proportions of young people in LMIC), problems with case finding (greater in LMICs), and differing overall mortality rates in the study base populations (usually higher in LMICs). Thus, the magnitude of the disparity suggested by SMRs may be deceptive, and it appears likely that the actual burden of premature mortality with epilepsy, if this were measured as incidence rates in entire populations, would be much higher in LMICs.

### Implications

A limited number of studies demonstrate substantially elevated risks of premature mortality among people with epilepsy in LMICs of three continents. Much of this may be attributed to the restricted distribution of healthcare resources in these countries, resources often far more limited than in HICs.^43^ Thus, lack of access and decreased adherence to medical management with AEDs place people with epilepsy at increased risk of fatal medical or injury-related complications of frequent seizures. Lack of access to prompt medical interventions for prolonged seizures places people with epilepsy at increased risk of death from status epilepticus. This mortality could be significantly reduced with improved access to health care including AEDs, and with education about the risks of epilepsy and ways to reduce these risks. In many communities, public education may be important to increase reliance on standard medical treatments of epilepsy as these are available, discourage its sole treatment by unproven folk medicine and traditional healing, and reduce its stigma.^46–48^ Also needed are improved public health and safety programs for primary prevention of structural causes of epilepsy—such as malaria, cysticercosis, and other infections of central nervous system—and accidents resulting in traumatic brain injuries.

The limited existing data on epilepsy-related mortality in LMICs are, however, not sufficient for optimum development of prevention programs adapted to local needs. Too many countries and local regions are not described. The circumstances of fatalities from medical complications, for example, status epilepticus, are uncertain. What proportion are associated with no AED treatment and in what proportion is AED use interrupted or reduced because of inability to afford its cost? The types and circumstances of fatal injuries arising from seizures are also uncertain. Where do drownings in people with epilepsy occur, how many are occupational, and which occupations are involved? How do fatal burns occur? How many traffic fatalities involve individuals with epilepsy as pedestrians, vehicle passengers, or vehicle drivers? How do local laws address motor vehicle driving by people with epilepsy? How many injuries involve falls from heights and how many of these are occupational? How do risks vary according to local resources, customs, and industries (e.g., climbing trees for fruit picking)? Although current LMIC data on epilepsy mortality broadly indicate the urgency of prevention programs now, future research can improve such programs by providing answers to questions like those above.

### Limitations

There are substantial limitations to these data. The studies that met our inclusion criteria represented only a small number of communities in Sub-Saharan Africa, Asia, and South America, whereas other major regions of the world, such as North Africa and the Middle East, were entirely unrepresented. Underrepresentation of major regions may be associated with limitations of economic resources affecting the capacity of their health care systems to implement prevention programs, manage seizures in people with epilepsy, and conduct epidemiologic studies of mortality in epilepsy. Healthcare systems and practices may also be affected independently by variations in regional culture, thus restricting our ability to generalize the findings from this review to unrepresented regions among LMICs.

The quality of the studies we reviewed varied considerably. The large heterogeneity observed illustrates the high degree of uncertainty in the estimation of the true occurrence of epilepsy-related mortality across LMICs. Incomplete ascertainment of epilepsy cases, a major concern in many of the studies, is likely to yield underestimates of the total burden of epilepsy mortality in general populations of LMICs. However, to the extent that studies overrepresent more severe forms of epilepsy that carry higher risk of premature death, comparative measurements of mortality such as SMR could be increased. Some population-based studies included only cases with convulsive epilepsies, whereas others may have incompletely ascertained cases with non-convulsive epilepsies. Most clinical cohorts overrepresented people with severe forms of epilepsy, such as one Brazilian study with 72% of enrollees who reported daily seizures^38^ and another that enrolled people with refractory epilepsy awaiting or having undergone epilepsy surgery.^36^ The diagnosis of epilepsy in many studies was not made by clinicians with training in the diagnosis of epilepsy, thus the accuracy of the estimates and causes of death in epilepsy in some studies should be viewed with caution. Most of the causes of death were ascertained by review of clinical records or verbal autopsy. The accuracy of verbal autopsies varies depending on the other common causes of death in the population, the tool used, and the experience of the person administering the tool.^23^ As for postmortem examinations, in HICs the proportion of deaths autopsied in general populations and in people with epilepsy can be low^49,50^; however, in comparison, none of the LMIC studies in this review included autopsy data.

More epidemiologic studies, involving more LMIC localities, are needed. As with future studies in HICs, studies in LMICs should be performed in conformity with current guidelines for epidemiologic studies.^15,17^ Representative population samples and incident cohorts should be studied, where acute symptomatic seizures are distinguished from single unprovoked seizures and from epilepsy, and where convulsive and nonconvulsive forms of epilepsy are also distinguished. Finally, epileptogenic conditions and all risk factors implicated in the mortality of epilepsy should be clearly described. The collection of such higher quality information will enable us to identify many measures necessary to prevent much of the premature mortality in LMICs.

## Supplementary Material

Supplementary tables

Supplementary figure 1

Supplementary figure 2

Supplementary figure 3

## Figures and Tables

**Table 1:** Mortality estimates from population-based studies

Population-based	Country-location (income)	Total population	Quality	People with epilepsy original cohort	People with epilepsy followed	Duration of FU (years)^a^	Estimated person-years	Deaths	SMR	95% CI	CFR	95% CI	Mortality rate per 1,000 person-year	95% CI
Ngugi (2014)^35^	Kenya-rural (L)	232,164	100	754	606	2.7	2,048	61	6.5	5.00–8.30	8.1	6.1–10	29.8	22.8–38.3
Kochen (2005)^8^	Argentina-urban (M)	70,000	90	106	96	8	768	8	2.45	1.14–4.65^a^	8.3	2.8–13.9	10.4	4.5–20.5
Kaiser (2007)^31^	Uganda-rural (L)	4,743	90	61	57	7	399	18	7.2	4.40–11.6	31.6	19.5–43.6	45.1	26.7–71.3
Nicoletti (2009)^34^	Bolivia-rural (M)	55,675	85	118	103	10	1,030	10	1.34	0.68–2.39	9.7	4.0–15.4	9.7	4.7–17.9
Houinato (2013)^30^	Benin-rural (L)	11,688	80	160	150	1.5	225	5			3.3	0.5–6.2	22.2	7.2–51.9
Banerjee (2010)^7^	India-urban (M)	52,377	80	337	337	5	1,685	20	2.58	1.50–4.13	5.9	3.4–8.5	11.9	7.3–18.3
Carpio (2005)^27^	India-rural (Vusai) (M)	16,000	80	51	51	10	510	10	3.9	2.1–7.25	19.6	8.7–30.5	19.6	9.4–36.1
Summary: higher quality population studies		442,647		1,587	1,400	5.8^b^	6,665	132	2.6^c^		8.1^c^		19.8	16.7–23.5
Carpio (2005)^27^	India-urban (Parsis) (M)	14,010	65	109	104	14	1,456	34	0.76	0.51–1.01	32.7	23.7–41.7	23.4	16.2–32.6
Kamgno (2003)^32^	Cameroon-rural (L)	NR	60	271	128	10	1,280	37			28.9	21.1–36.8	28.9	20.4–39.8
Mu (2011)^33^	China-rural (M)	5,840,000	50	3,568	2,998	4.5	13,491	106	4.9	4.0–6.1	3.5	2.9–4.2	7.9	6.4–9.5
Ding (2013)^29d^	China-rural (M)	3,185,000	50	2,455	1,986	6.1	12,114	206	2.9	2.6–3.4	10.4	9.0–11.7	17.0	14.8–19.5
Carpio (2005)^27^	Mali-urban/rural (L)	7,158	40	36	31	12	372	13	4.25	2.8–6.45	41.9	24.6–59.3	34.9	18.8–59.8
Summary: lower quality population studies		>9,112,561		8,894	7,233	6.0^b^	32,850	431	2.9^c^		10.4^c^		13.1	11.9–14.4

FU, follow-up; SMR, standardized mortality ratio; CI, confidence interval; CFR, case fatality ratio; (L), low-income country; (M), middle-income country.

a Length of follow-up described variously as median (Ngugi et al.^35^), mean (Ding et al.^29^), or the total interval of cohort assessment (others).

b Mean weighted by study person-years.

c Median weighted by study person-years.

d Follow-up study of cohort described in Ding et al.^28^

**Table 2:** Mortality estimates from clinical cohort studies

	Country-location	Quality	People with epilepsy in original cohort	People with epilepsy followed	Duration of FU (years)	Person-years	Deaths	SMR	95% CI	Case fatality ratio (%)	95% CI	Mortality rate per 1,000 person-year	95% CI
Carpio (2005)^27^	Ecuador-urban (M)	50	420	379	3	1,137	7	6.3	2.0–10.0	1.8	0.5–3.2	6.2	2.5–12.7
Almeida (2010)^36^	Brazil-urban/Rural (M)	45	550	550	10	5,500	16			2.9	1.5–4.3	2.9	1.7–4.7
Terra (2011)^38^	Brazil-urban (M)	35	1,012	987	10	9,870	53			5.4	4.0–6.8	5.4	4.0–7.0
Jilek-Aall (1992)^12^	Tanzania-Rural (L)	35	164	146	30	4,380	110			75.3	68.4–82.3	25.1	20.6–30.3
Thomas (2001)^41^	India-urban (M)	25	447	246	12	2,952	18			7.3	4.1–10.6	6.1	3.6–9.6
Terra (2010)^39^	Brazil-urban/Rural (M)	25	267	267	13	3,471	9			3.4	1.2–5.5	2.6	1.2–4.0
Devilat (2004)^37^	Chile-urban/Rural (M)	5	NR	NR	6	NR	16	3.21	1.5–5.0				
Terra (2009)^40^	Brazil-urban/Rural (M)	5	996	835	8	6,680	11			1.3	0.5–2.1	1.6	0.8–2.9
Summary: all studies			3,856	3,410	12.4^a^	33,990	240	4.8		5.4^b^		7.1	6.2–8.0

FU, follow-up; SMR, standardized mortality ratio; CI, confidence interval; CFR, case fatality ratio; (L), low-income country; (M), middle-income country.

a Mean weighted by study person-years.

b Median weighted by study person-years.

**Table 3:** Estimates of proportional mortality in epilepsy by cause in population-based and clinical cohort studies

Study	Country-location	Design	Quality (%)	Number of people with epilepsy followed	Number of deaths	Causes of death (%)			
						Direct	Indirect	Unrelated	Undetermined
Ngugi (2014)^35^	Kenya-rural	Population	100	606	61	44.3	11.4	34.3	9.8
Kaiser (2007)^31^	Uganda-rural	Population	90	61	18	33.0	17.0	44.4	5.6
Nicoletti (2009)^34^	Bolivia-rural	Population	85	103	10	10	20	50	20
Banerjee (2010)^7^	India-urban	Population	80	337	20	5	30	45	20
Kamgno (2003)^32^	Cameroon-rural	Population	60	271	37	75.5	10.8	13.7	
Mu (2011)^33^	China-rural	Population	50	2,998	106	21.6	58.8	19.6	
Ding (2013)^29^	China-rural	Population	50	1,986	206	14.1	32.5	39.3	14.1
Carpio (2005)^27^	Mali-rural/urban	Population	40	36	13	38.0	NR	62.0	NR
Summary: all population-based				6,546	471	27.3^a^	20.0^a^	41.9^a^	14.1^a^
Carpio (2005)^27^	Ecuador-urban	Clinical cohort	50	379	7	42	30	28	
Almeida (2010)^36^	Brazil-urban/rural	Clinical cohort	45	550	16			62.5	
Terra (2011)^38^	Brazil-urban	Clinical cohort	35	987	53	15.1		84.9	
Jilek-Aall (1992)^12^	Tanzania-rural	Clinical cohort	35	164	110	17.3	18.2	32.7	31.8
Terra (2010)^39^	Brazil-urban/ rural	Clinical cohort	25	267	9	77.8			
Devilat (2004)^37^	Chile-Santiago	Clinical cohort	5	NR	16	39.3			
Summary: all clinical cohorts				>2,347	211	39.3^a^	24.1^a^	47.6^a^	

a Median percentage.

## Key Points

- Mortality in people with epilepsy in low- and middle-income countries (LMICs) is estimated to be 2.6-fold higher (range 1.3–7.2) than in general populations of LMICs
- The main causes of death in people with epilepsy in LMICs are epilepsy-related complications, that is, status epilepticus, SUDEP, and injuries
- Estimates of the public health burden of epilepsy-related mortality are higher in LMICs than estimates from high-income countries
- Insufficient studies of good quality, high heterogeneity, and underrepresentation of diverse LMIC economies and culture were the main limitations of this systematic review
- Improving access to medical facilities and antiepileptic drugs, and preventing injuries may substantially reduce premature mortality in epilepsy in LMICs
