# Supplementary material for: Premature mortality of epilepsy in low- and middle-income countries: A systematic review from the Mortality Task Force of the International League Against Epilepsy
Source: Epilepsia. Author manuscript; Available in PMC 2020 Feb 11. (PMC7012644; doi:10.1111/epi.13603)
Supplement: Supplementary tables [file EMS85643-supplement-Supplementary_tables.docx]

**Supporting Information**

**Supporting Table 1A. Criteria and grading for assessing qualities of mortality studies**

| Sensitivity of Epilepsy Case Ascertainment (20/20)   - 20/20 = Screening methods appear likely to ascertain nearly all (≥ 85%) cases in population - 15/20 = Screening methods appear likely to ascertain most (70 - 84%) cases in population - 10/20 = Screening methods appear likely to ascertain majority (50 - 69%) of cases in population - 5/20 = Screening methods appear unlikely to ascertain majority of cases in population OR information published is insufficient to assess - 0/20 N/A = Not applicable: not a population-based study or sensitivity of methods of epilepsy case ascertainment not relevant to quality of study.   *Sensitivity of Mortality Case Ascertainment (20/20)*   - 20/20 = Fatalities appear likely to be recorded in nearly all (≥ 85%) cases in study population - 15/20 = Fatalities appear likely to be recorded in most (70 - 84%) cases in study population - 10/20 = Fatalities appear likely to be recorded in majority (50 - 69%) of cases in study population - 5/20 = Fatalities appear unlikely to be recorded in majority of cases in study population OR information published is insufficient to assess   *Accuracy of Diagnoses of Epilepsy (20/20)*   - 20/20 = Cases are diagnosed (or confirmed) by specialist clinician (i.e., with neurologic training), AND ILAE case definition applied - 15/20 = Cases are often diagnosed by non-specialist clinician, OR minor deviation from ILAE case definition - 10/20 = All or substantial proportion of cases diagnosed based on self-report or non-clinical sources with specified criteria judged to have fair positive predictive value - 5/20 = All or substantial proportion of cases diagnosed with poorly defined criteria from non-clinical sources; positive predictive value judged to be poor OR information published is insufficient to assess   *Accuracy of Diagnoses of Cause of Death (20/20)*   - 20/20 = Determined mainly from either autopsy, ME/coroner investigation, or other clinical investigation (e.g., review of medical records, structured interview of survivors or “verbal autopsy”) - 15/20 = Determined largely or wholly from death certificate data, when such data are judged to have good positive predictive value for the specific causes of interest - 10/20 = Determined largely or wholly from death certificate data, when such data are judged to have only fair positive predictive value for the specific causes of interest - 5/20 = Other sources of data deemed to have poor positive predictive value for the causes of interest OR information published is insufficient to assess - 0/20 = Cause of death not studied   Representativeness of the study population (20/20)   - 20/20 = Cohort studies of incident epilepsy whose enrolled cases appear highly representative of the population of interest - 15/20 = Studies of prevalent epilepsy whose enrolled cases appear highly representative of the population of interest - 10/20 = Studies of epilepsy whose enrolled cases appear somewhat representative of the population of interest - 5/20 = Studies of epilepsy whose enrolled cases appear poorly representative of the population of interest or where representativeness cannot be assessed. |
| --- |

Supporting Table 1B: Standard definition of epidemiological indicators of mortality in epilepsy

**Case fatality Ratio** is the proportion of people with epilepsy in a cohort who die, usually expressed as a percentage.

**Proportionate mortality Rate (PMR)** is the ratio of the number of deaths due to a specific cause in a population to the total number of deaths in the same period.

**Mortality rate** in epilepsy is the number of deaths (all causes) in a population of individuals diagnosed with epilepsy people with epilepsy, and is calculated from number of deaths per person years of observation.

**Age-specific mortality fraction** is the number of deaths in a given age group per time, usually expressed per 1,000 or 100,000 persons per year.

**Cause specific mortality rate** is mortality rate from a specified cause for a population during a specified time period usually expressed per 1,000 or 100,000 persons per year.

**Standardized mortality ratio** **(SMR)** is a [ratio](https://ibis.health.state.nm.us/resources/Glossary.html#R) between the observed number of deaths in people with epilepsy and the number of deaths that would be expected, based on the age- and sex-specific [rates](https://ibis.health.state.nm.us/resources/Glossary.html#R) in a standard population a. If the ratio of observed/expected deaths >is greater than 1.0, there is said to be "excess deaths" in the study population. With respect to the mortality in epilepsy, this measure is used to compare mortality in epilepsy with mortality in general population.

Supporting Table 2: Summary of characteristics and quality of population-based studies of mortality in epilepsy

| Study | Country-Location | | Dates | Source | Population  (denominator) | Epilepsy cases | Case ascertainment % | Diagnosis | Mortality captured % | Cause  of Death | Representativeness | Overall Quality  (Max 100) |
| --- | --- | --- | --- | --- | --- | --- | --- | --- | --- | --- | --- | --- |
| Kochen 2005[^8^](#_ENREF_8) | Argentina-Urban | | 1991-1998 | PBS | 70,000 | 106 | ≥85 | ILAE by  Neurologist | ≥85 | DC | Highly | 90 |
| Houinato 2013[^30^](#_ENREF_30) | Benin- Rural | | 2006-2007 | PBS +  MR+ KI | 11,688 | 160 | ≥85 | ILAE by  Neurologist | ≥85 | N/A | Highly | 80 |
| Nicoletti 2009[^34^](#_ENREF_34) | Bolivia-Rural | | 1994-2004 | PBS | 55,675 | 118 | ≥85 | ILAE | ≥85 | NR | Highly | 85 |
| Kamgno 2003[^32^](#_ENREF_32) | Cameroon- Rural | | 1991-2001 | KI | NR | 271 | 50-69 | ILAE by  Physicians | 70-84 | NR | Somewhat | 60 |
| Ding 2006[^28^](#_ENREF_28) | | China-Rural | 2000-2004 | PHC | 3,185,000 | 2455^†^ | N/A | NR | 70-84 | VA | Somewhat | 50 |
| Mu 2011[^33^](#_ENREF_33) | | China-Rural |  | PHC | 5,840,000 | 3568^†^ | N/A | NR | 70-84 | VA | Somewhat | 50 |
| Ding 2013[^29^](#_ENREF_29) | | China-Rural | 2000-2008 | PHC | 3,185,000 | 2455^†^ | N/A | NR | 70-84 | VA+DC | Somewhat | 50 |
| Carpio 2005[^27^](#_ENREF_27) | India- Rural | | 1985-1999 | PBS | 14,010 | 109 | ≥85 | Neurologist | ≥85 | N/A | Poorly | 65 |
| Banerjee 2010[^7^](#_ENREF_7) | India- Urban | | 2003-2005 | PBS | 52,377 | 337 | ≥85 | ILAE by  Neurologist | ≥85 | N/A | Highly | 80 |
| Carpio 2005[^27^](#_ENREF_27) | India- Urban | | 1989-1994 | PBS | 16,000 | 51^†^ | ≥85 | NR | ≥85 | VA | Somewhat | 80 |
| Ngugi 2014^30^ | Kenya- Rural | | 2007-2010 | PBS | 232,164 | 754^†^ | ≥85% | ILAE by  Neurologist | ≥85 | VA | Highly | 100 |
| Carpio 2005[^27^](#_ENREF_27) | Mali-Urban, Rural | | 1988-2000 | KI | 7,158 | 36^†^ | 50 - 69 | NR | 70-84 | NR | Poorly | 40 |
| Kaiser 2007[^31^](#_ENREF_31) | Uganda- Rural | | 1994-2001 | PBS | 4,743 | 61 | ≥85 | ILAE | ≥85 | VA | Somewhat | 90 |

Key: DC Clinical Diagnosis; KI: Key informants, MR: Medical records, N/A: Not Available; NR Not Recorded; PBS: Population-based screening, PHC: Primary health care, VA Verbal Autopsy,

ILAE: International League Against Epilepsy

*These studies describe the same cohort.

^†^Convulsive epilepsy only.

Supporting Table 3: Summary of characteristics and quality of clinical cohort studies of mortality in epilepsy

| Study | Country-Location | Dates | Source of clinical cohort | Epilepsy cases | Case | Diagnosis | Mortality | Cause  of Death | Representativeness | Overall Quality  (Max 100) |
| --- | --- | --- | --- | --- | --- | --- | --- | --- | --- | --- |
| Carpio 2005[^27^](#_ENREF_27) | Ecuador-Urban | 1997-2000 | Tertiary Hospital | 420 | N/A | ILAE | ≥85 | N/A | Somewhat | 50 |
| Almeilda 2010[^36^](#_ENREF_36) | Brazil-Both | 1992-2002 | Tertiary Hospital | 550 | N/A | Clinical | ≥85 | N/A | Poor | 45 |
| Terra 2011[^38^](#_ENREF_38) | Brazil-Urban | 2000-2010 | Tertiary Hospital | 1012 | N/A | N/R | ≥85 | SR | Poor | 35 |
| Jilek-Aall 1992[^12^](#_ENREF_12) | Tanzania-Rural | 1960-1990 | District Hospital | 164 | N/A | N/R | ≥85 | N/A | Somewhat | 35 |
| Thomas 2001[^41^](#_ENREF_41) | India-Urban | 1985-1997 | Tertiary Hospital | 447 | N/A | N/R | 50 - 69 | N/A | Somewhat | 25 |
| Terra 2010[^39^](#_ENREF_39) | Brazil-Both | 1995-2008 | Tertiary Hospital | 267 | N/A | N/R | > 80 | N/I | Poor | 25 |
| Terra 2009[^40^](#_ENREF_40) | Brazil-Both | 2000-2008 | Tertiary Hospital | 996 | N/A | N/R | N/R | N/I | Poor | 5 |
| Devilat 2004[^37^](#_ENREF_37) | Chile-Both | 1996-2002 | Tertiary Hospital |  | N/A | N/R | N/R | N/I | Poor | 5 |

Key: N/A – Not Available; N/R – Not recorded, ILAE- International League Against Epilepsy

**Supporting Table 4: Estimates of proportionate mortality ratio (PMR) from SUDEP and status epilepticus among people with epilepsy**

|  |  |  | **PMR in %** | |
| --- | --- | --- | --- | --- |
| **Study** | **Country-Location** | **Quality** | **SUDEP** | **SE** |
| *Population based studies* | | | | |
| Ngugi 2014[^35^](#_ENREF_35) | Kenya-Rural | 100 | 6.6^a^ | 37.7^a^ |
| Kaiser 2007[^31^](#_ENREF_31) | Uganda-Rural | 90 | 11.1 | 22.2 |
| Carpio 2005[^27^](#_ENREF_27) | India-Urban-Vasai | 80 | 20 ^a^ |  |
| Nicoletti 2009[^34^](#_ENREF_34) | Bolivia-Rural | 85 |  | 10 |
| Banerjee 2010[^7^](#_ENREF_7) | India-Urban | 80 |  | 5 |
| Kamgno 2003[^32^](#_ENREF_32) | Cameroon-Rural | 60 | 18.9 | 56.6 |
| Mu 2011[^33^](#_ENREF_33) | China, Rural | 50 | 14.7^b^ | 6.9 |
| Ding 2013[^29^](#_ENREF_29) | China, Rural | 50 | 1^b^ | 13.1 |
| Median All Population Studies | |  | 12.9 | 13.1 |
| *Clinical cohort studies* | | | | |
| Carpio 2005[^27^](#_ENREF_27) | Ecuador, Urban | 50 | 14.3 ^c^ | 23 |
|  |  |  | 28.6 ^b^ |  |
| Almeida 2010[^36^](#_ENREF_36) | Brazil, Urban-Rural | 45 | 2.9 ^b^ |  |
| Terra 2011[^38^](#_ENREF_38) | Brazil, Urban | 35 | 13.2 | 15.1 |
| Jilek-Aall 1992[^12^](#_ENREF_12) | Tanzania, Rural | 35 |  | 14.5 |
| Terra 2010[^39^](#_ENREF_39) | Brazil, Urban-Rural | 25 | 11.1 |  |
| Devilat 2004[^37^](#_ENREF_37) | Chile-Santiago | 5 | 31.25 ^b^ |  |
|  | Urban/Rural |  | 6.25 ^c^ |  |
| Terra 2009[^40^](#_ENREF_40) | Brazil-Urban, Rural | 5 | 1.38 |  |
| Median All clinical cohorts | |  | 11.1 | 14.8 |

Key: PMR: Proportionate mortality ratio, SUDEP: Sudden death in epilepsy, SE: Status epilepticus

^a^Cases described as possible.

^b^Cases described as probable.

^c^Cases described as definite.

**Supporting Table 5: Estimates of proportionate mortality ratio (PMR) in epilepsy by type of injury**

|  | |  | |  |  | PMR by type of injury | | | | |
| --- | --- | --- | --- | --- | --- | --- | --- | --- | --- | --- |
| Study | | Country-Location | | Quality | Falls | | Drowning | Traffic injury | Burns | Suicide |
| *Population based studies* | | | | | | | | | | |
| Ngugi 2014[^35^](#_ENREF_35) | Kenya-Rural | | 100 | | 3.3 | | 3.3 | 1.6 | 1.6 |  |
| Kaiser 2007[^31^](#_ENREF_31) | Uganda-Rural | | 90 | |  | | 5.6 |  | 11.1 |  |
| Nicoletti 2009[^34^](#_ENREF_34) | Bolivia-Rural | | 85 | |  | |  | 10 |  | 10 |
| Banerjee 2010[^7^](#_ENREF_7) | India-Urban | | 80 | |  | | 15 | 15 |  |  |
| Kamgno 2003[^32^](#_ENREF_32) | Cameroon-Rural | | 60 | |  | | 10.8 |  |  |  |
| Ding 2006[^28^](#_ENREF_28) | China, Rural | | 50 | |  | | 37 | 2.9 |  |  |
| Mu 2011[^33^](#_ENREF_33) | China, Rural | | 50 | | 5.9 | | 45.1 | 4.9 |  | 2.9 |
| *Clinical cohort studies* | | | | | | | | | | |
| Terra 2011[^38^](#_ENREF_38) | | Brazil, Urban | | 35 | 1.9 | | 1.9 |  |  |  |
| Jilek-Aall 1992[^12^](#_ENREF_12) | | Tanzania, Rural | | 35 |  | | 12.7 |  | 5.5 |  |

Key: PMR: Proportionate mortality ratio

Supporting Table 6: Estimates of causes of death reported in SMR and MR

| Study | Country-Location | Quality | Measure | Causes | Estimate (95% C.I.) |
| --- | --- | --- | --- | --- | --- |
| Mu 2011[^33^](#_ENREF_33) | China, Rural | 50 | SMR | Cerebrovascular | 1.14 (0.36–3.61) |
|  |  |  |  | Cardiac disease | 1.60 (0.50–5.22) |
|  |  |  |  | Influenza and pneumonia | 1.05 (0.26–4.32) |
|  |  |  |  | Malignant neoplasm | 1.94 (0.90–4.18) |
|  |  |  |  | Other diseases | 11.41 (4.03–32.43) |
| Ding 2013[^29^](#_ENREF_29) | China, Rural | 50 | SMR | Drowning | 39.0 (26.4–55.5) |
|  |  |  |  | Toxic effects | 17.0 (6.9–35.7) |
|  |  |  |  | Falls | 9.8 (3.6–21.7) |
|  |  |  |  | Suicide | 8.2 (4.5–14.0) |
|  |  |  |  | Transport injury | 6.0 (2.8–11.4) |
|  |  |  |  | Myocardial infarction | 3.6 (1.6–7.2) |
|  |  |  |  | Digestive system diseases | 4.4 (2.3–7.7) |
|  |  |  |  | Pneumonia | 2.9 (0.7–7.8) |
|  |  |  |  | Cerebrovascular | 2.2 (1.5–3.1) |
|  |  |  |  | Neoplasms | 1.1 (0.7–1.8) |
| Terra 2009[^40^](#_ENREF_40) | Brazil, Urban-Rural | 5 | MR | Unrelated to epilepsy | 22.2 |

**Key:** SMR-Standardized mortality ratio, MR-Mortality rate, CI-Confidence interval

**Supporting Table 7: Proportionate Mortality Ratio (PMR) by Type of Seizure**

|  |  |  |  | PMR for epilepsy type | | |
| --- | --- | --- | --- | --- | --- | --- |
| Study | Location | Source | Quality | Generalized | Focal | Unknown |
| Kochen 2005[^8^](#_ENREF_8) | Argentina-Urban | Population | 90 | 37.5 | 50 | 12.5 |
| Nicoletti 2009[^34^](#_ENREF_34) | Bolivia-Rural | Population | 85 | 40 | 60 |  |
| Almeida 2010[^36^](#_ENREF_36) | Brazil, Urban-Rural | Clinical Cohort | 45 | 14.3 | 85.7 |  |
| Thomas 2001[^41^](#_ENREF_41) | India, Urban | Clinical Cohort | 25 | 38.9 | 61.1 |  |

Key: PMR-Proportionate mortality ratio (%)

Supporting Table 8: Estimates of mortality in epilepsy by seizure frequency

| Study | Location | Source | Quality | Measure | Frequency | SMR/MR |
| --- | --- | --- | --- | --- | --- | --- |
| Kaiser 2007[^31^](#_ENREF_31) | Uganda-Rural | Population | 90 | SMR | High  (>1 per week) | 14.7  (8.5—24.8) |
|  |  |  |  |  | Low  (<1 per week) | 1.4  (0.4—5.7) |
| Terra 2009[^40^](#_ENREF_40) | Brazil- Urban/Rural | Cohort | 5 | MR | Daily | 0.63 |
|  |  |  |  |  | 2-4 per week | 0.50 |
|  |  |  |  |  | 1 per month | 0.13 |

Key: SMR-Standardized mortality ratio, MR-Mortality rate

**Supporting Table 9: Estimates of mortality in epilepsy by duration of epilepsy in population-based studies**

| Study | Country-Location | Quality | Measure | Duration of epilepsy (years) | MR/SMR |
| --- | --- | --- | --- | --- | --- |
| Ngugi 2014[^35^](#_ENREF_35) | Kenya-Rural | 100 | MR | <1 | 45.9(22.9–91.7) |
|  |  |  |  | 1–5 | 30.8(19.1–49.5) |
|  |  |  |  | 6–10 | 35.9(20.4–63.2) |
|  |  |  |  | >10 | 31.1(20.9–46.4) |
| Kaiser 2007[^31^](#_ENREF_31) | Uganda-Rural | 90 | SMR | 0-4 | 8.6 (4.5-16.5) |
|  |  |  |  | 5-9 | 3.6 (1.1-11.4) |
|  |  |  |  | 10-14 | 23.8 (8.9-65.5) |

Key: SMR-Standardized mortality ratio, MR-Mortality rate

Supporting Table 10: Estimates of proportionate mortality ratios (PMR) in epilepsy by etiology

|  |  |  |  | Etiology | | | |
| --- | --- | --- | --- | --- | --- | --- | --- |
| Study | Country-Location | Quality | Measure | Cryptogenic | Symptomatic | Remote seizure | Undetermined |
| Almeida 2010[^36^](#_ENREF_36) | Brazil, Urban-Rural | 45 | PMR | 21.4 | 78.6 |  |  |
| Devilat 2004[^37^](#_ENREF_37) | Chile-Urban/Rural | 5 | PMR |  | 81.3 |  |  |
| Kochen 2005[^8^](#_ENREF_8) | Argentina-Urban | 95 | PMR |  |  | 75 | 25 |
| Nicoletti 2009[^34^](#_ENREF_34) | Bolivia-Rural | 85 | PMR |  |  | 60 |  |
|  |  |  | SMR | 0.74  (0.2–1.8)* |  | 3  (1.2–6.3) |  |

Key: PMR-Proportionate mortality rate, SMR-Standardized mortality rate

***** Idiopathic

Supporting Table 11: Estimates of mortality in epilepsy by treatment

| Study | Country-Location | Quality | Measure | Treatment | Result |
| --- | --- | --- | --- | --- | --- |
| Ngugi 2014[^35^](#_ENREF_35) | Kenya-Rural | 100 | MR | Adherence | 16.1 (9.5–27.2) |
|  |  |  |  | Non adherence | 48.8 (36.7–65.0) |
| Kaiser 2007[^31^](#_ENREF_31) | Uganda-Rural | 90 | SMR | Good adherence | 7.4 |
|  |  |  |  | Poor adherence | 8.0 |
| Kamgno 2003[^32^](#_ENREF_32) | Cameroon-Rural | 60 | PMR | AED | 13.6 |
|  |  |  |  | AED + Traditional | 27.3 |
| Mu 2011[^33^](#_ENREF_33) | China, Rural | 50 | PMR | Phenobarbital  Dose: 30–60 mg. | 47.2 |
|  |  |  |  | Phenobarbital  Dose: 90–180 mg. | 44.3 |
|  |  |  |  | Phenobarbital  Dose: 210–240 mg. | 8.5 |
| Nicoletti 2009[^34^](#_ENREF_34) | Bolivia-Rural |  | PMR | Treatment in past year | 20 |
|  |  |  |  | No treatment in past year | 80 |
|  |  |  |  | Treatment in last month | 20 |
|  |  |  |  | No treatment in last month | 80 |

Key: AED-Antiepileptic drug, MR-Mortality rate, SMR-Standardized mortality ratio, PMR-Proportionate mortality ratio

**Supporting Figure 1**: **SMR and PMR of epilepsy by sex**

[Figure submitted in separate file]

Key: P=Parsis, V=Vusai. Panel A and B compare mortality rate by sex as reported through SMR and PMR. SMR stands for standardized mortality ratio; ratio of age standardized mortality rate in epilepsy and general population. SMR greater than 1 represent excess mortality in epilepsy than general population. PMR stands for proportionate mortality ratio; a ratio of deaths attributed to epilepsy and all deaths in the population. The ratio is presented as percent of epilepsy deaths in the population.

**Supporting Figure 2: Forest plot of excess mortality in epilepsy by quality of studies**

[Figure submitted in separate file]

Key: CI-Confidence interval, V-Vusai, P-Parsis, M-Mali, E-Ecuador

Forest plot present a graphical representation of a meta-analysis stratified by quality of studies. X-axis represent standardized mortality ratio (SMR), a ratio of age standardized mortality rate in epilepsy and general population. The plot summarizes individual and combined estimates of SMR as indicated by shaded-black dots and diamond-shaped box respectively. I-V Subtotal/Overall = fixed effects meta-analysis and D + L Subtotal/Overall = random effect meta-analysis. Study weights were estimated based on standard error of individual studies. I-square present a statistical test of heterogeneity within high and low quality studies and between group (high vs. low quality studies).

**Supporting Figure 3: Forest plot of excess mortality in epilepsy by duration of cohort follow-up**

[Figure submitted in separate file]

Key: CI-Confidence interval, V-Vusai, P-Parsis, M-Mali, E-Ecuador

Forest plot present a graphical representation of a meta-analysis stratified by duration cohort follow-up. X-axis represent standardized mortality ratio (SMR), a ratio of age standardized mortality rate in epilepsy and general population. The plot summarizes individual and combined estimates of SMR indicated by shaded black dots and diamond shaped box. I-V Subtotal/Overall = fixed effects meta-analysis and D + L Subtotal/Overall = random effect meta-analysis. Study weights were estimated based on standard error of individual studies. I-square present a statistical test of heterogeneity within high and low quality studies and between group (high vs. low quality studies).
