# Supplementary figures and images for: Premature mortality of epilepsy in low- and middle-income countries: A systematic review from the Mortality Task Force of the International League Against Epilepsy
Source: Epilepsia. Author manuscript; Available in PMC 2020 Feb 11. (PMC7012644; doi:10.1111/epi.13603)

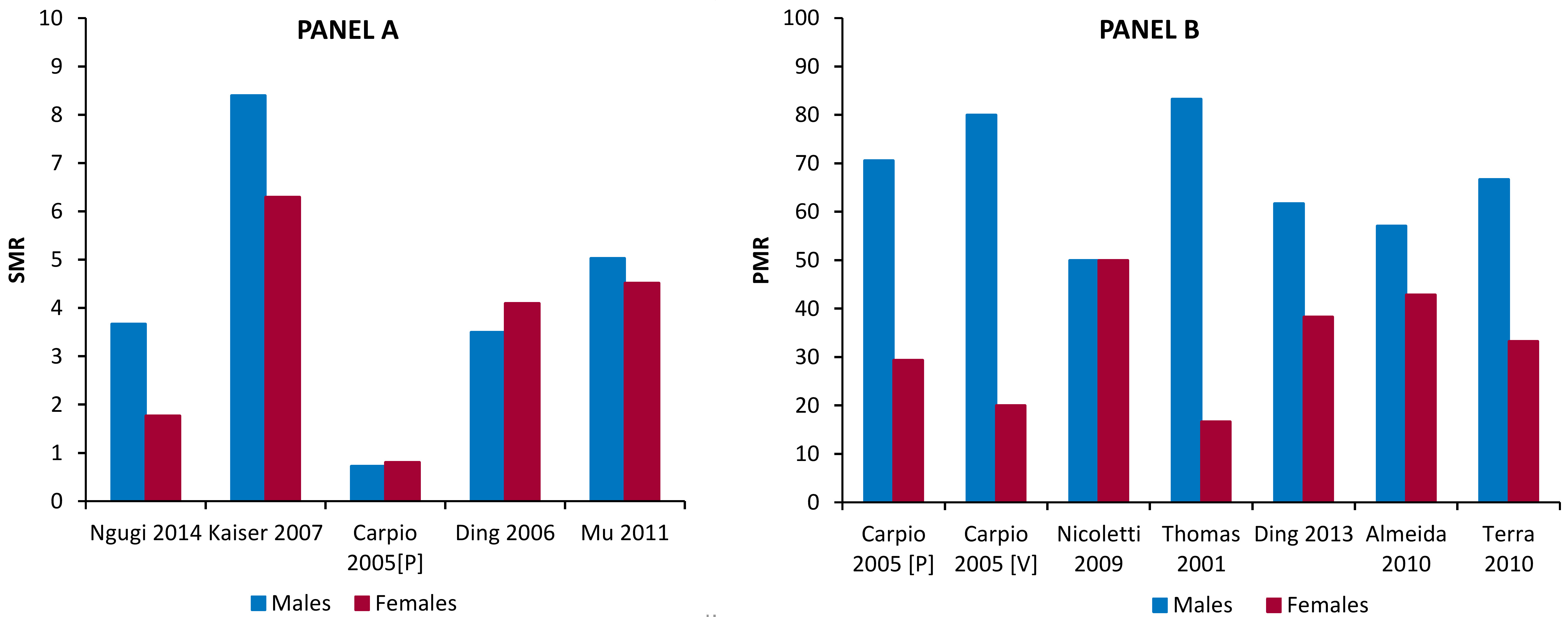

Supplement: Supplementary figure 1 [file EMS85643-supplement-Supplementary_figure_1.tif]

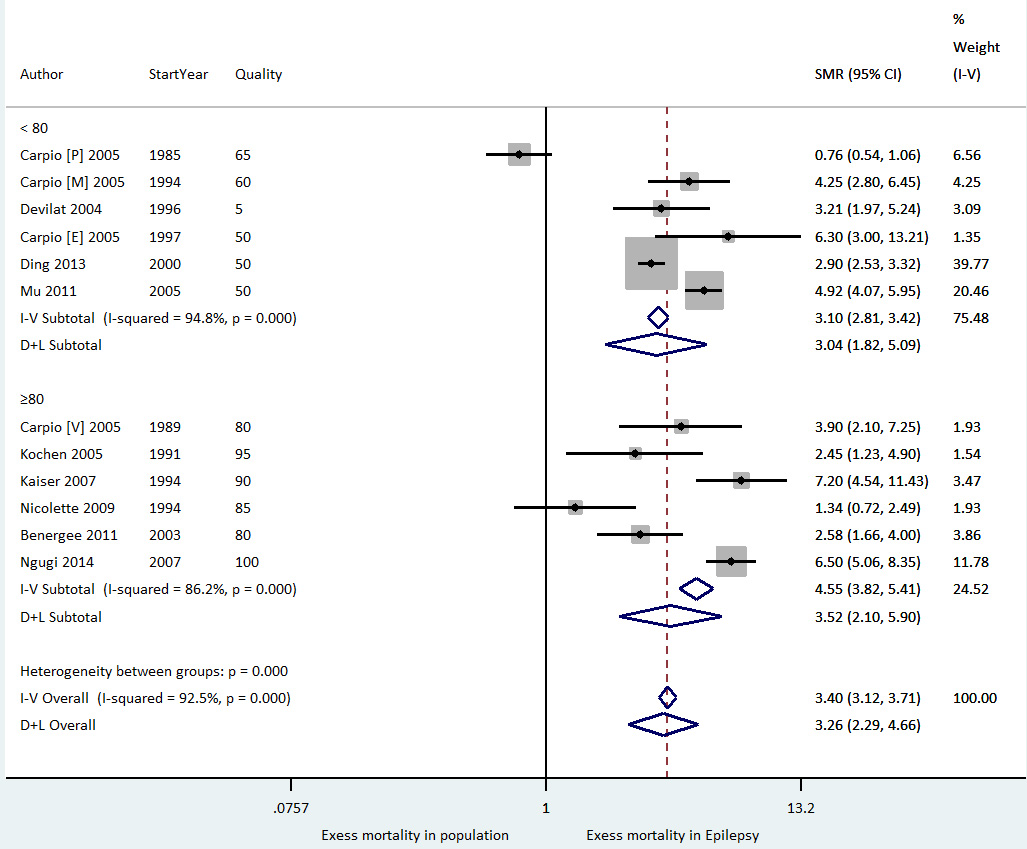

Supplement: Supplementary figure 2 [file EMS85643-supplement-Supplementary_figure_2.tif]

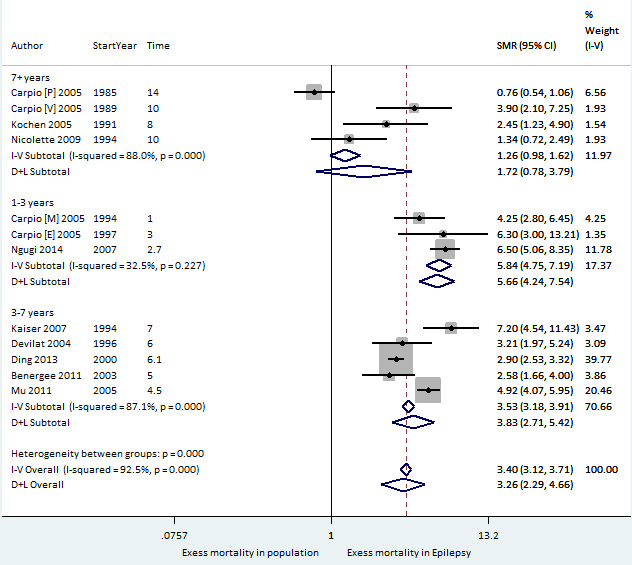

Supplement: Supplementary figure 3 [file EMS85643-supplement-Supplementary_figure_3.tif]
